# Unsupervised mobile app-based cognitive testing in a population-based study of older adults born 1944

**DOI:** 10.3389/fdgth.2022.933265

**Published:** 2022-11-08

**Authors:** Fredrik Öhman, David Berron, Kathryn V. Papp, Silke Kern, Johan Skoog, Timothy Hadarsson Bodin, Anna Zettergren, Ingmar Skoog, Michael Schöll

**Affiliations:** ^1^ Department of Psychiatry and Neurochemistry, Institute of Neuroscience and Physiology, Sahlgrenska Academy, University of Gothenburg, Gothenburg, Sweden; ^2^ Wallenberg Centre for Molecular and Translational Medicine, University of Gothenburg, Gothenburg, Sweden; ^3^ German Center for Neurodegenerative Diseases (DZNE), Magdeburg, Germany; ^4^ Clinical Memory Research Unit, Department of Clinical Sciences Malmö, Lund University, Lund, Sweden; ^5^ Center for Alzheimer’s Research and Treatment, Department of Neurology, Brigham and Women’s Hospital, Harvard Medical School, Boston, MA, United States; ^6^ Department of Neurology, Massachusetts General Hospital, Harvard Medical School, Boston, MA, United States; ^7^ Department of Psychology, University of Gothenburg, Gothenburg, Sweden; ^8^ Dementia Research Centre, Queen Square Institute of Neurology, University College London, London, United Kingdom

**Keywords:** Alzheimers disease (AD), digital cognitive assessment, smartphone-based cognitive assessments, remote and unsupervised assessment, episodic memory

## Abstract

**Background:**

Mobile app-based tools have the potential to yield rapid, cost-effective, and sensitive measures for detecting dementia-related cognitive impairment in clinical and research settings. At the same time, there is a substantial need to validate these tools in real-life settings. The primary aim of this study was thus to evaluate the feasibility, validity, and reliability of mobile app-based tasks for assessing cognitive function in a population-based sample of older adults.

**Method:**

A total of 172 non-demented (Clinical Dementia Rating 0 and 0.5) older participants (aged 76–77) completed two mobile app-based memory tasks—the Mnemonic Discrimination Task for Objects and Scenes (MDT-OS) and the long-term (24 h) delayed Object-In-Room Recall Task (ORR-LDR). To determine the validity of the tasks for measuring relevant cognitive functions in this population, we assessed relationships with conventional cognitive tests. In addition, psychometric properties, including test-retest reliability, and the participants’ self-rated experience with mobile app-based cognitive tasks were assessed.

**Result:**

MDT-OS and ORR-LDR were weakly-to-moderately correlated with the Preclinical Alzheimer's Cognitive Composite (PACC5) (*r* = 0.3–0.44, *p* < .001) and with several other measures of episodic memory, processing speed, and executive function. Test-retest reliability was poor–to-moderate for one single session but improved to moderate–to-good when using the average of two sessions. We observed no significant floor or ceiling effects nor effects of education or gender on task performance. Contextual factors such as distractions and screen size did not significantly affect task performance. Most participants deemed the tasks interesting, but many rated them as highly challenging. While several participants reported distractions during tasks, most could concentrate well. However, there were difficulties in completing delayed recall tasks on time in this unsupervised and remote setting.

**Conclusion:**

Our study proves the feasibility of mobile app-based cognitive assessments in a community sample of older adults, demonstrating its validity in relation to conventional cognitive measures and its reliability for repeated measurements over time. To further strengthen study adherence, future studies should implement additional measures to improve task completion on time.

## Introduction

Identifying individuals with cognitive impairment at risk of developing Alzheimer's disease (AD) *via* conventional in-clinic cognitive assessment is time-consuming and costly. Conventional cognitive testing is limited with regards to test frequency and design as it requires trained staff and the individual on-site for each assessment. Recent technological developments have created the opportunity to implement mobile app-based cognitive testing on smartphones and tablets, both in population screening and clinical assessment settings. Digital and unsupervised cognitive assessments could thus serve as pre-screening in the health care system before more expensive and invasive clinical examinations such as cerebrospinal fluid sampling or neuroimaging are performed. Furthermore, they could represent more reliable and scalable cognitive baseline measures and outcomes in clinical trials, allowing for remote assessment of a larger population than samples obtained *via* in-clinic and supervised examinations (1–4).

Trends in digitalization suggest that older adults are increasingly familiar and comfortable with new technologies, and surveys from 2019 indicate that 77% of North Americans aged 50+ own a smartphone (5). In the European Nordic countries, 78% of people born in Sweden during the 1940s (74–84-year-olds) use smartphones, and 43% use tablets regularly. 83% of Sweden's older adults born during the 1940s use the Internet (66% use it daily). In this survey, 100% report that they have access to the Internet in their household (6).

While there is also potential in various novel assessment techniques such as passive monitoring (7) (e.g., using sensors and wearables), spoken language analysis and automated language processing (8), eye tracking (9), digital pens (e.g., Digital Clock Drawing) (10), this study solely focuses on unsupervised mobile app-based cognitive testing. Mobile app-based cognitive testing can address some of the shortcomings of conventional in-clinic cognitive testing. For example, automatically alternating test versions avoid or diminish practice effects (11, 12) and enable more frequent and flexible cognitive testing (13). More frequent assessments may also increase reliability in cognitive measurement (14, 15). Furthermore, it may yield more sensitive disease-related measures (16, 17), such as accelerated forgetting (18), and the possibility of employing tests of long-term delayed recall.

However, several potential challenges remain unexplored as mobile app-based testing is performed in an uncontrolled environment (1, 2). First, the feasibility of mobile app-based examinations in older adults, including examining the psychometric properties (15, 19–21), participant experience (15, 19, 22), and external aspects of testing (16). Second, participant adherence to unsupervised and remote study designs is an important research question (23). Finally, there are many important and so far understudied aspects that might affect task performance in unsupervised study settings. Thus, we will explore contextual factors such as potential distractions, device-specific factors such as screen size, and individual traits including age and education.

We employed two mobile app-based cognitive tasks building on recent findings on the functional brain architecture of episodic memory and the spatiotemporal progression of AD pathology (24–26). First, the Mnemonic Discrimination Task for Objects and Scenes (MDT-OS) (27), taxing pattern separation as a short-term memory task (28). Pattern separation is the process of discriminating among highly similar but unique pieces of information (e.g., where you parked your car today vs. yesterday). Second, the Object-In-Room Recall Task (ORR-LDR) was developed to tax pattern completion (29), i.e., the ability to retrieve a stored memory based on a cue of incomplete information. The ORR-LDR was implemented as a one- to three-day long-term delayed recall task, consequently assessing long-term memory.

In this study, we investigated how demographic and contextual factors affect task performance in these tasks in a population-based sample of individuals aged 76–77, and how the participants rated their experience using the app. There is still little research on the psychometric properties of mobile app-based cognitive testing. Therefore, we investigated the psychometric properties of this type of cognitive assessment, including reliability over repeated sessions (test-retest reliability) (30). We also investigated the score distribution of the tasks due to the importance that a cognitive test has sufficient range of scores to detect individual differences in performance (floor or ceiling effects) (31). Finally, we assessed how well the employed app-based tests correlated with conventional in-clinic cognitive tests (construct validity), including gold-standard neuropsychological testing commonly used as cognitive measures in clinical practice, such as the optimized version of the Preclinical Alzheimer Cognitive Composite (PACC5) (32), designed to be sensitive to early AD-related cognitive impairment. Given that the tasks evaluated in this study are primarily memory tasks, we hypothesized that they would be associated most strongly with other measures of memory (convergent validity) and less so with measures that theoretically should not be highly related (discriminant validity).

## Materials and methods

### Sample

The Gothenburg H70 Birth Cohort Studies are longitudinal population-based studies of health and aging. The most recent cohort examined includes 1,203 individuals born in 1944 (559 men and 644 women) (33). Using the Swedish Tax Agency's population register, individuals born in 1944 (mean age = 70.5 years) were invited to participate in the baseline in-clinic examination (January 2014–December 2016).

**Figure 1:**
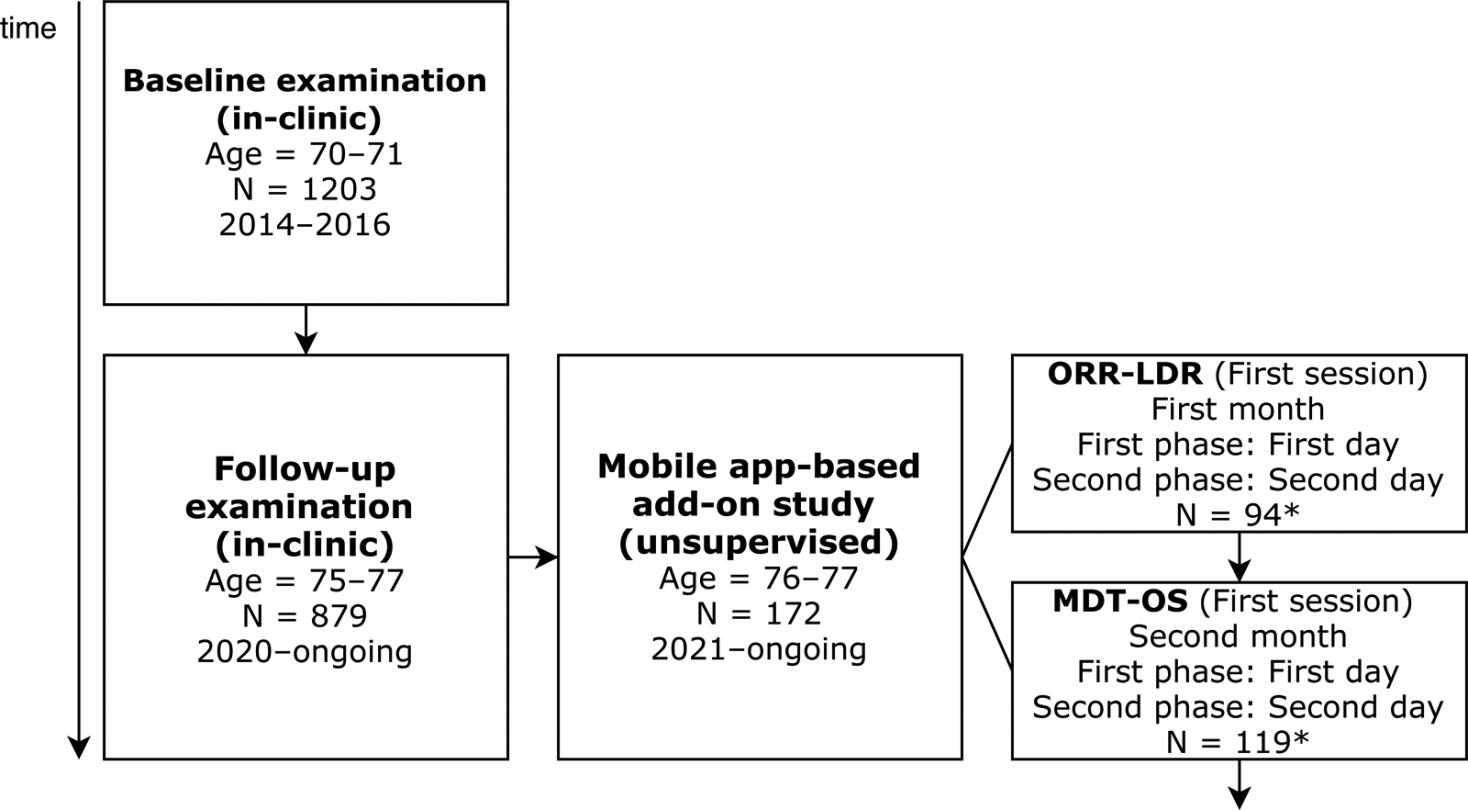
Flowchart of baseline and follow-up examinations. ORR-LDR, Object-in-Room Recall Task; MDT-OS, Mnemonic Discrimination Task for Objects and Scenes. *Please note, these numbers represent participants that correctly completed all phases after initial filtering (see section 3.1).

The five-year follow-up in-clinic examination was initiated in 2020. Due to the COVID-19 pandemic, participants are still being invited for follow-up assessments according to the study protocol (see Figure 1 for a flowchart of the study design). At the time of this study, 879 participants have completed their follow-up in-clinic examination. The clinical and neuropsychological assessments included in this study were from the follow-up examination initiated in 2020. In parallel with the follow-up study, we have invited participants to the mobile app-based add-on study (which will be described in the following section).

### Remote mobile app-based add-on study

In the course of the follow-up in-clinic examination, 692 of 879 participants have thus far been invited to participate in a two-year study using the neotiv-App (12, 34). Of these, 172 participants have signed the informed consent and initiated the mobile app-based cognitive testing to date. The remainder of the participants will be invited to participate in future recruitment waves.

Each month's testing consisted of a two-phase session separated by 24 h. For ORR-LDR, the two phases were encoding (ORR encoding, lasting ∼10 min) and long-term delayed recall (ORR-LDR, lasting ∼4 min). For MDT-OS, the phases (lasting ∼10 min each) were two halves of the mnemonic discrimination task (one-back and two-back memory task, respectively). All test sessions were completed by participants remotely and unsupervised, following a Bring-Your-Own-Device (BYOD) approach. During the mobile app-based study, participants were contacted *via* telephone and offered help to install the app correctly when needed. Besides this contact, there was no further personal contact with the participants.

Here, we primarily present the results of the first test session of each task. Every month, the participants were reminded *via* push notifications to initiate their monthly tasks. Even though participants were recommended to do the tasks at a given time of the day, there was no restriction on when the task could be initiated. The very first test session could be initiated at any time of the day. Precisely 24 h after the end of the first test session, a push notification was sent to initiate the second test session. Before each session, participants were informed by the app to carry out the task in a quiet environment, to ensure that they had their glasses ready if needed and that their screen was bright enough to see the stimuli well. They also underwent a short practice session before the actual task session. Following each task session, participants were asked to state whether they were distracted by anything in their surrounding environment and to rate their concentration, subjective self-performance, task interest, and task effort. All written communication, including app instructions, was in Swedish language.

### Mobile app-based test measures

#### Object-In-Room recall task

**Figure 2:**
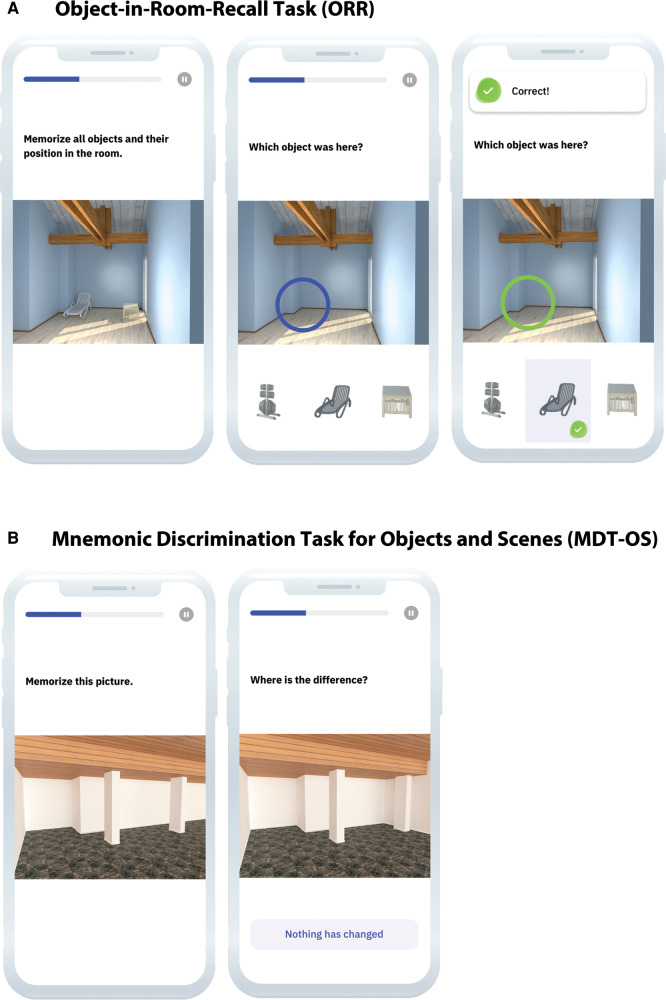
(**A**) ORR-LDR and (**B**) MDT-OS. ORR-LDR, Object-in-Room Recall Task; MDT-OS, Mnemonic Discrimination Task for Objects and Scenes. Used with permission from neotiv GmbH.

In ORR-LDR, participants see a computer-generated room with two 3D-rendered objects (see Figure 2) (12, 34). In the encoding phase, participants learn the position of two objects in a room and are tested for immediate retrieval. During the immediate retrieval, they see empty rooms and are asked to recall which object was at a specific location. The aim is to encode 25 object and scene associations. They respond *via* tapping on one of three objects pictured below the empty room (one is the correct object for that location, the second is the object presented in the room but at a different location, and the last is an entirely unrelated object). Twenty-four hours later, the same memory recall task is repeated. The primary outcome of this task for our study is the total amount of recalled objects after 24 h (ranging between 0 and 25).

#### Mnemonic discrimination task for objects and scenes

In MDT-OS, participants are shown 3D-rendered computer-generated objects and scenes, repeated either identically or slightly altered (see Figure 2) (24, 27). Here the task is to determine whether a replicated display is a repetition of the original stimulus or an altered version. An answer is given by tapping on the location of the difference (for stimuli believed to be altered). If no change is perceived, the button “nothing has changed” is clicked. In total, participants are shown 32 object and 32 scene pairs. Half of the pairs are identical or altered, respectively. The whole task is separated into two phases and conducted during two days with a 24-hour delay. The first phase is designed as a one-back task, while the second phase is designed as a two-back task. In this study, the outcome is the corrected hit rate. Hit rate (trials where identically repeated stimuli are identified correctly) and a false alarm rate (trials where slightly altered stimuli are incorrectly identified as identical repetitions) are calculated and subtracted from each other (hit rate minus false alarm rate). The possible scores range between 0 and 1.

### Clinical and neuropsychological assessments

Participants underwent the follow-up clinical examinations at the Sahlgrenska University Hospital Memory Clinic in Mölndal, Sweden, or in their homes. The examinations were performed on average 11.16 months before the mobile app-based study (SD = 6.48 months; range = 0.34–21.49 months).

Research nurses administered the neuropsychological examinations primarily *via* a tablet (iPad Pro 12.9″, 3rd generation) using the Delta cognitive testing platform (35). Our neuropsychological examinations comprised tests for (a) speed and attention: Digit span (36), Trail making test A (TMT A) (37), (b) memory: Rey Auditory Verbal Learning Test (RAVLT) (38), Brief Visuospatial Memory Test—Delta version (BVMT) (39), (c) executive function: Digit-Symbol-Test—Delta version (Digit-Symbol-Test) (36), Category fluency test animals (Word fluency animals) (40), Controlled Oral Word Association Test FAS (Word fluency FAS) (41), Trail making test B (TMT B) (37), Stroop—Delta version (Stroop) (42) and (d) visuospatial function: Block Design—SRB 3 version (Block Design) (43). For all digitalized tests, designs were equivalent or similar to paper-and-pencil counterparts. For tests where a pencil traditionally functions as an input (TMT A/B, Digit-Symbol-Test, Stroop, and BVMT), the Apple Pencil (2nd generation) was used.

The PACC (32) is a cognitive composite developed to be sensitive to AD-related cognitive impairment and consists of episodic memory, executive function, and global cognition measures. We created a PACC5 (44) cognitive composite using an average of z-scored measures of RAVLT delayed recall (included twice), MMSE, Digit-Symbol-Test, and Word fluency animals. PACC weighs heavily on aspects of episodic memory, and PACC5 additionally incorporates elements of semantic memory, i.e., category fluency.

None of the participants had a dementia diagnosis at baseline (age = 70) as defined by the diagnostic and statistical manual of mental disorders, 3rd ed., revised (DSM–III–R) criteria. The MMSE (45) was used to measure global cognitive function, and the Clinical Dementia Rating (CDR) (46) to measure clinical dementia staging. At follow-up, 152 participants had a CDR score of 0 (cognitively normal), and 20 had a score of 0.5 (questionable dementia). The CDR is based on six areas of cognitive and functional performance (memory, orientation, judgment and problem solving, community affairs, home and hobbies, and personal care). No participant had CDR 1 (mild dementia), CDR 2 (moderate dementia), or CDR 3 (severe dementia).

### Statistical analyses

Linear regressions were used to determine the relationships between demographics (sex and education), contextual factors (distractions), device-specific factors (screen size), self-rated experience (self-reported task performance, task concentration, task interest, and task effort) and mobile app-based cognitive measures. Group differences were assessed using independent samples Welch's *t*-tests, Student's *t*-tests, and *χ*^2^ test. The relationships between the mobile app-based cognitive measures and conventional neuropsychological tests were assessed using Spearman correlation. Test-retest reliability was calculated using intraclass correlation coefficient (ICC) (30) of individual sessions. We also calculated ICC based on mean-rating (for an average of two sessions) (*k* = 2), absolute-agreement, mixed-effects model. ICC was reported alongside their 95% confident intervals. All tests were two-tailed, and a *p*-value <0.05 was considered statistically significant. All statistical analyses were performed using R Statistical Software (version R 4.1.2) (47). Recruitment for the study is ongoing. Here we analyzed the data collected up until March 1, 2022.

## Results

### Recruitment, adherence, and filtering

We included study participants who completed at least one test session of the two cognitive tasks (each session consisting of two phases). As one complete test session was scheduled per month, at least two months were needed to collect the data. A total of 64% of the participants used smartphones and 36% tablets; 41% used android OS and 59% iOS devices. To date, 172 participants have signed the informed consent and initiated the mobile app-based cognitive testing, of which 100% have finished the first phase of the first month, 92% (*n* = 158) have completed all phases for the first month of testing, 75% (*n* = 129) have finished the first phase of the second month of testing, and 69% (*n* = 119) have completed the second phase of the second month of testing. As this is an ongoing study, and participants have been informed that it is acceptable to perform the test sessions even after the scheduled time point, we cannot yet draw any final conclusions on participant attrition (e.g., drop-outs).

In order to address potential recruitment bias, we performed group comparisons between those that signed the informed consent and initiated the mobile app-based cognitive testing (*n* = 172) and those that have not yet chosen to participate (*n* = 520). The groups did not differ in terms of sex (*p* = .102) and CDR (*p* = .790). While years of education (*p* = .043) and MMSE (*p* = .002) did significantly differ between the groups, the mean differences were only minimal (0.16 difference in MMSE score and 0.74 difference in education years). Thus, there was a very small effect where the group that did enroll in the mobile app study was slightly better educated and had a minimally higher global cognitive function.

In the final dataset, we filtered the data according to the following criteria. For ORR-LDR, 35% (*n* = 56) of the participants performed the delayed recall after a longer time than allowed (72 h). We decided to exclude these sessions from further analyses because the time between encoding and retrieval (time-to-retrieval) was negatively associated with task performance (*β* = −0.15, *p* < .001, 95% CI, −0.21, −0.10), and these data might obfuscate the results. The proportion of participants exceeding the restriction for time-to-retrieval was smaller in participants with CDR 0.5 than those with CDR 0. For participants with CDR 0.5, only 25% were excluded. Furthermore, a chi-square test of independence showed no significant association between CDR score (0 vs. 0.5) and correct vs. incorrectly performed time-to-retrieval. Additionally, we restricted the number of timeouts (e.g., not responding to a given trial in time) as technical problems or other issues cannot be ruled out. The minimum percentage of answered trials was set to 60% of total task items. This restriction resulted in the further exclusion of eight participants for ORR-LDR and none for MDT-OS.

The final dataset consisted of 94 (CDR 0 *N* = 81; CDR 0.5 = 13) participants that successfully completed the ORR-LDR and 119 (CDR 0 *N* = 102; CDR 0.5 = 17) participants that successfully completed the MDT-OS.

### Clinical measures

Demographics and descriptive statistics for mobile app-based and conventional neuropsychological measures are described in Table 1. *T*-tests comparing these measures of participants with CDR 0 and CDR 0.5 showed that the groups did not differ on any of the measures besides MMSE score (*p* = .021), MDT-OS (*p* = .040), and Word fluency FAS (*p* = .039) where participants with CDR 0 showed slightly better performance.

**Table 1:** Demographics and cognitive measures.

Demography	Total (*n* = 172)	CDR 0 (*n* = 152)	CDR 0.5 (*N* = 20)	CDR 0 vs. 0.5 (*p-*value)
Sex (Male/Female)	88/84	80/72	8/12	0.760
Age	77.05 (0.30)	77.04 (0.31)	77.17 (0.15)	
Age range	76–77	76–77	76–77	
Education	14.13 (3.82)	14.15 (3.75)	13.93 (4.42)	0.801
Cognitive measures				
Mobile app-based				
ORR-LDR (*n* = 94^a^)	11.93 (3.44)	11.90 (3.26)	12.08 (4.54)	.836
MDT-OS (*n* = 119^a^)	0.38 (0.16)	0.39 (0.16)	0.31 (0.13)	.040*
Global				
PACC5 composite	0.00 (0.68)	0.03 (0.66)	−0.27 (0.81)	.065
MMSE	29.09 (1.21)	29.22 (1.00)	28.10 (1.97)	.021*
Speed and attention				
TMT A	40.94 (14.45)	40.23 (15.38)	46.30 (15.25)	.099
Digit span	15.37 (4.26)	15.36 (4.30)	15.35 (4.04)	.981
Memory				
RAVLT encoding	38.50 (9.30)	38.81 (9.26)	36.15 (9.56)	.231
RAVLT delayed recall	6.82 (3.40)	6.91 (3.41)	6.15 (3.28)	.347
BVMT encoding	16.54 (6.15)	16.46 (6.38)	17.04 (4.71)	.756
BVMT delayed recall	7.45 (2.54)	7.44 (2.67)	7.50 (1.52)	.913
Visuospatial				
Block Design	21.46 (6.70)	21.76 (6.77)	19.50 (6.10)	.161
Executive				
Digit-Symbol-Coding	50.68 (11.83)	50.76 (11.44)	50.75 (14.69)	.977
Word fluency animals	22.19 (5.60)	22.26 (5.69)	21.65 (4.92)	.647
TMT B	96.08 (39.50)	93.83 (35.39)	113.10 (61.04)	.182
Word fluency FAS	45.20 (14.47)	46.03 (14.23)	38.95 (15.08)	.039*
Stroop	36.29 (10.76)	36.53 (10.67)	34.55 (11.50)	.442

Results are presented as mean and SD, unless otherwise stated. Independent samples *t*-test was used for significance testing, except for sex, where chi-square test was used.

ORR-LDR, Object-in-Room Recall Task; MDT-OS, Mnemonic Discrimination Task for Objects and Scenes; PACC5, Preclinical Alzheimer's Cognitive Composite; MMSE, Mini-Mental State Examination; RAVLT, Rey Auditory Verbal Learning Test; TMT, Trail Making Test; BVMT, Brief Visuospatial Memory Test—Delta version.

^a^ These numbers represent participants that correctly completed all phases after initial filtering (see section 3.1).

* *p* < .05.

### Relationship to demographics

The associations between the mobile app-based cognitive measures and sex and years of education were investigated using multiple linear regression models. Years of education was not associated with task performance on any of the tasks (ORR encoding, *β* = 0.02, *p* = .790, 95% CI, −0.10, 0.14; ORR-LDR, *β* = 0.08, *p* = .315, 95% CI, −0.08, 0.26; MDT-OS phase 1, *β* = 0.00, *p* = .072, 95% CI, −0.00, 0.02; MDT-OS phase 2, *β* = 0.00, *p* = .407, 95% CI, −0.01, 0.01). Likewise, sex was not associated with task performance on any of the tasks (ORR encoding, *β* = −0.39, *p* = 0.402, 95% CI, −1.31, 0.53; ORR-LDR, *β* = 1.02, *p* = .153, 95% CI, −0.38, 2.43; MDT-OS phase 1, *β* = −0.00, *p* = .963, 95% CI, −0.07, 0.07; MDT-OS phase 2, *β* = −0.02, *p* = .497, 95% CI, −0.09, 0.04).

### Self-rated task experience

Participants' experiences using mobile app-based cognitive tasks in a remote unsupervised setting give insight into the acceptance and tolerability of these novel measures. Table 2 outlines the full details of the self-rated task experience. Directly following each test phase, the participants were asked to report whether they had been distracted and to rate their subjective concentration, task performance, task interest, and task effort. Few (an average of 10%) participants reported that they were distracted by something in their environment. A clear majority (an average of 95%) stated that their concentration was average or better. Self-rated task interest was good, with very few (an average of 5%) participants rating the tasks uninteresting. Questions regarding self-rated task effort indicated that a minority (22%) found the encoding phase of ORR-LDR demanding. In comparison, 57% found the long-term delayed recall phase demanding. A clear majority (an average of 78%) deemed both phases of the MDT-OS demanding. Again, a clear majority (89%) rated their performance as average or better for the encoding phase of ORR-LDR, while only 47% did for the long-term delayed recall. For MDT-OS, 59% rated their performance as average or better for the first phase, while 39% did so for the more challenging second phase.

**Table 2:** Self-reported measures.

Self-reported task disturbance					
	Not distracted during task			Distracted during task	
ORR encoding	89%			11%	
ORR-LDR	90.4%			9.6%	
MDT-OS (phase 1)	89.9%			10.1%	
MDT-OS (phase 2)	89.9%			10.1%	
Self-reported task concentration					
	Very bad	Bad	Average	Good	Very good
ORR encoding	0%	2.3%	23.3%	51.7%	22.7%
ORR-LDR	0%	6.4%	34%	44.7%	14.9%
MDT-OS (phase 1)	0.8%	1.5%	24.8%	48.8%	24%
MDT-OS (phase 2)	1.7%	3.4%	21%	53.8%	20.2%
Self-reported task performance					
	Very bad	Bad	Average	Good	Very good
ORR encoding	0.6%	9.9%	30.8%	43%	15.7%
ORR-LDR	7.4%	45.74%	38.3%	8.5%	0%
MDT-OS (phase 1)	8.5%	32.6%	47.3%	11.6%	0%
MDT-OS (phase 2)	15.7%	45.4%	34.5%	4.2%	0%
Self-reported task interest					
	Very uninteresting	Uninteresting	Neither	Interesting	Very interesting
ORR encoding	0.6%	1.7%	12.2%	64%	21.5%
ORR-LDR	0%	4.3%	19.1%	64.9%	11.7%
MDT-OS (phase 1)	0.8%	3.1%	22.5%	58.9%	14.7%
MDT-OS (phase 2)	1.7%	5.9%	22.7%	58.8%	10.9%
Self-reported task effort					
	Very demanding	Demanding	Neither	Undemanding	Very undemanding
ORR encoding	0%	21.5%	27.9%	37.2%	13.4%
ORR-LDR	10.6%	46.8%	35.1%	6.4%	1.1%
MDT-OS (phase 1)	19.4%	51.9%	24%	3.9%	0.8%
MDT-OS (phase 2)	28.6%	55.5%	15.1%	0.8%	0%

Note that percentages may not equal 100% because of rounding.

ORR-LDR, Object-in-Room Recall Task; MDT-OS, Mnemonic Discrimination Task for Objects and Scenes.

Self-rated task concentration was not significantly associated with task performance on ORR-LDR (*β* = 0.86, *p* = .052, 95% CI, −0.00, 1.72) and MDT-OS (phase 1: *β* = 0.04, *p* = .087, 95% CI, −0.00, 0.08; phase 2: *β* = 0.02, *p* = .361, 95% CI, −0.02, 0.06). Self-rated task performance was significantly positively associated with task performance on MDT-OS phase 1 (*β* = 0.06, *p* = .003, 95% CI, 0.02, 0.10), but not ORR-LDR (*β* = 0.84, *p* = .081, 95% CI, −0.11, 1.79) and MDT-OS phase 2 (*β* = 0.03, *p* = .239, 95% CI, −0.02, 0.07). Self-rated task interest was not significantly associated with task performance on ORR-LDR (*β* = 1.14, *p* = .344 95% CI, −1.25, 3.54) and MDR-OS (phase 1: *β* = 0.10, *p* = .078, 95% CI, −0.01, 0.21; phase 2: *β* = 0.11, *p* = .058, 95% CI, −0.00, 0.22). Self-rated task effort was significantly positively associated with task performance on MDR-OS phase 1 (*β* = 0.04, *p* = .036, 95% CI, 0.00, 0.09), but not with ORR-LDR (*β* = 0.10, *p* = .829, 95% CI, −0.78, 0.98) and MDR-OS phase 2 (*β* = 0.04, *p* = .163, 95% CI, −0.01, 0.09).

### Distribution of test results

**Figure 3:**
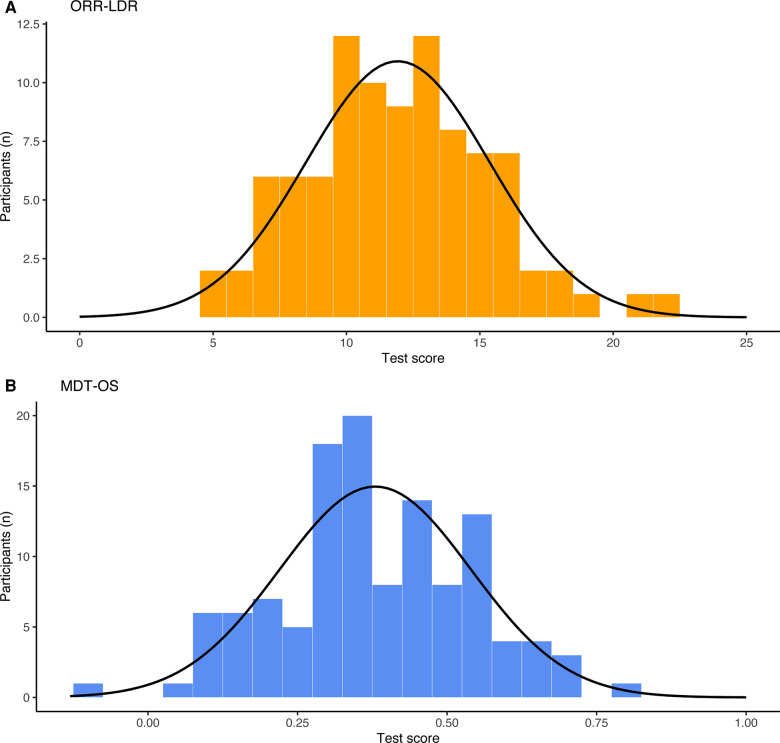
Distribution of test scores across (**A**) ORR-LDR and (**B**) MDT-OS. ORR-LDR, Object-in-Room Recall Task; MDT-OS, Mnemonic Discrimination Task for Objects and Scenes.

We examined the basic psychometric properties of each phase of the mobile app-based tasks, which is important given that a task's application area is both to detect subtle impairment in preclinical AD and clinical groups with more pronounced cognitive impairment. Floor and ceiling effects were defined by participants obtaining either the minimum or maximum score of a task. While we observed an expected ceiling effect in the ORR encoding phase, there were no apparent floor or ceiling effects in the delayed recall phase and neither in the MDT-OS. However, in ORR-LDR, 10% (*n* = 9) of participants scored below chance following a delay period of up to 72 h. Except for ORR encoding, all tasks were normally distributed (skewness = −0.07–0.32, kurtosis = 2.84–3.04). The skewness of the ORR encoding (skewness = −1.80, kurtosis = 6.53) can be attributed to the nature of the encoding phase, which is meant to ensure that the items have been encoded successfully. See Figure 3 for the distribution of test results across the outcome measures of the mobile app-based tasks.

### Test-retest reliability

The test-retest reliability of unsupervised tasks is relatively unexplored. It becomes important when considering the longitudinal cognitive trajectory of participants, regardless of whether the application of the specific task is a single measurement or the average across multiple measurements. Test-retest reliability measured by ICC using single sessions (*n* = 35) was poor for ORR-LDR when time-to-retrieval was restricted to a maximum of 72 h (see Table 3) (range: ICC 0.23–0.44; Session one vs. session two [ICC = 0.23, 95% CI, (−0.12, 0.52)], session one vs. session three (ICC = 0.44, 95% CI, 0.12, 0.67), session one vs. session four (ICC = 0.26, 95% CI, −0.05, 0.54), session two vs. session three (ICC = 0.33, 95% CI, 0.00, 0.595), session two vs. session four (ICC = 0.36, 95% CI, 0.05, 0.61), session three vs. session four (ICC = 0.33, 95% CI, 0.00, 0.59). For MDT-OS, test-retest reliability using single sessions (*n* = 53) was moderate [range: ICC 0.48–0.66; Session one vs. session two (ICC = 0.48, 95% CI, 0.24, 0.67)], session one vs. session three (ICC = 0.57, 95% CI, 0.35, 0.72), session one vs. session four (ICC = 0.51, 95% CI, 0.29, 0.69), session two vs. session three (ICC = 0.62, 95% CI, 0.34, 0.78), session two vs. session four (ICC = 0.52, 95% CI, 0.29, 0.70), session three vs. session four (ICC = 0.66, 95% CI, 0.45, 0.80).

**Table 3:** Psychometric properties.

Psychometric properties	ORR-LDR	MDT-OS
Ceiling effect (percentage)	0%	0%
Floor effect (percentage)	0%	0%
Skewness	0.32	−0.07
Kurtosis	3.04	2.84
Range	5–22	−0.09–0.78
SD	3.44	0.16
ICC (single sessions)	0.23–0.44	0.48–0.66
ICC (average of two sessions)	0.70	0.82

SD, standard deviation; ORR-LDR, Object-in-Room Recall Task; MDT-OS, Mnemonic Discrimination Task for Objects and Scenes.

In contrast, ICC using the average of two sessions yielded increased test-retest reliability—moderate for ORR-LDR (ICC = 0.70, 95% CI, 0.41, 0.85) and good for MDT-OS (ICC = 0.82, 95% CI, 0.70, 0.90)

### Influence of contextual and device-specific factors

In an unsupervised test setting, it is of interest how contextual and device-specific factors affect task performance. Using multiple linear regression models, we explored how the presence of distractions during tasks and device screen size were associated with task performance. The presence of distractions was associated with poorer performance for the encoding phase of ORR-LDR [*β* = −1.94, *p* = .042, 95% CI (−3.09, −0.20)]. The other tasks were not associated with the presence of distractions (ORR-LDR, *β* = −0.75, *p* = .571, 95% CI, −3.39, 1.88; MDT-OS phase 1, *β* = 0.10, *p* = .078, 95% CI, −0.21, 0.01; MDT-OS phase 2, *β* = −0.11, *p* = 0.058, 95% CI, −0.22, 0.00). Screen size was not associated with task performance on any of the tasks (ORR encoding, *β* = 0.02, *p* = .575, 95% CI, −0.06, 0.10; ORR-LDR, *β* = 0.00, *p* = .915, 95% CI, −0.12, 0.13; MDT-OS phase 1, *β* = 0.00, *p* = .933, 95% CI, −0.00, 0.00; MDT-OS phase 2, *β* = −0.00, *p* = 0.900, 95% CI, −0.00, 0.00).

### Validation against conventional cognitive measures

**Figure 4:**
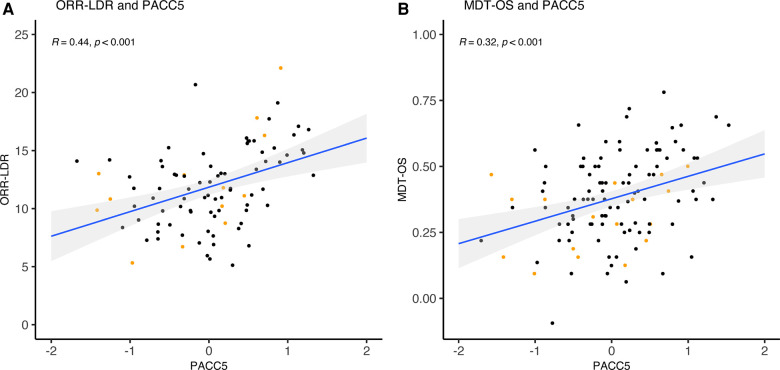
ORR-LDR, MDT-OS and correlation with PACC5. (**A**) ORR-LDR and (**B**) MDT-OS were weakly to moderately correlated with PACC5 (*r* = 0.44, *p* < .001, and *r* = 0.32, *p* < .001). The correlation values represent Spearman's Rho. Colored dots represent Clinical Dementia Rating (CDR) 0 (black) and CDR 0.5 (orange). ORR-LDR, Object-in-Room Recall Task; MDT-OS, Mnemonic Discrimination Task for Objects and Scenes; PACC5, Preclinical Alzheimer's Cognitive Composite.

**Figure 5:**
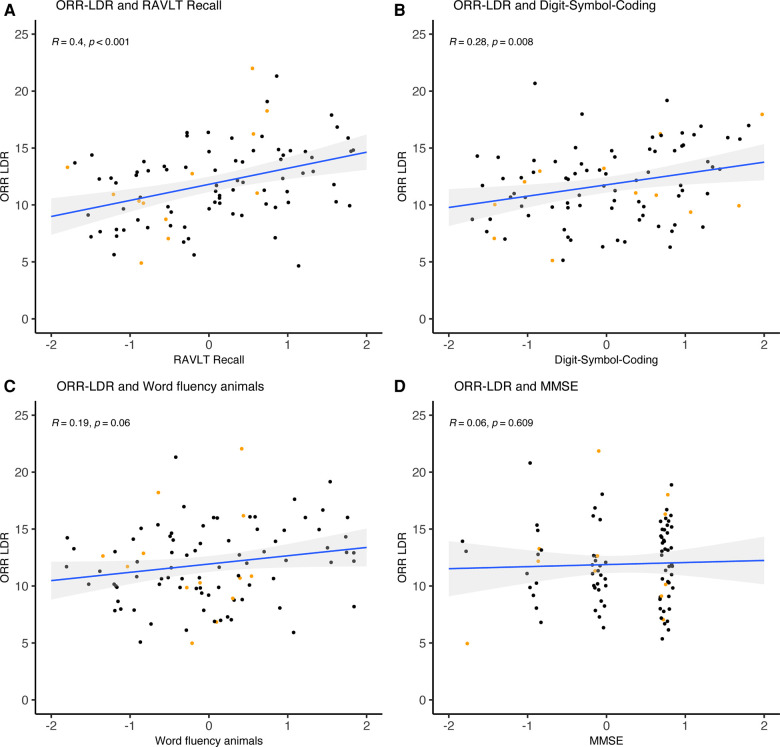
ORR-LDR and correlation with PACC5 subtests. ORR-LDR were weakly to moderately correlated with PACC5 subtests (**A**) RAVLT delayed recall (*r* = 0.40, *p* < .001) and (**B**) Digit-Symbol-Coding (*r* = 0.28, *p* = 0.008). ORR-LDR was not significantly correlated with the PACC5 subtests (**C**) Word fluency animals and (**D**) MMSE. The correlation values represent Spearman's Rho. Coloured dots represent Clinical Dementia Rating (CDR) 0 (black) and CDR 0.5 (orange). ORR-LDR, Object-in-Room Recall Task; RAVLT, Rey Auditory Verbal Learning Test; MMSE, Mini-Mental State Examination; PACC5, Preclinical Alzheimer's Cognitive Composite.

**Figure 6:**
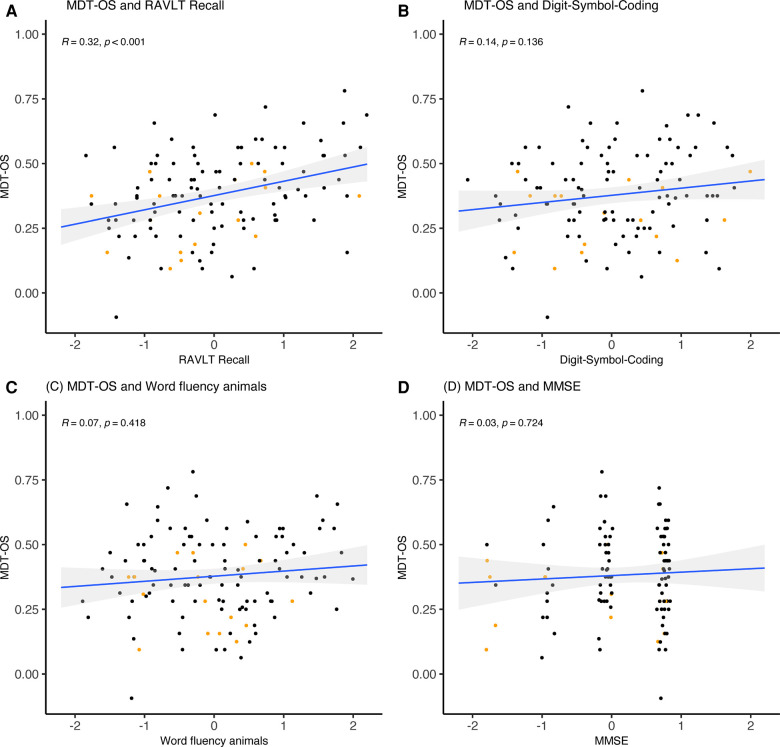
MDT-OS and correlation with PACC5 subtests. (**A**) MDT-OS was weakly correlated with the PACC5 subtest RAVLT Recall (*r* = 0.32, *p* < .001). MDT-OS was not significantly correlated with the PACC5 subtests (**B**) Digit-Symbol-Coding, (**C**) Word fluency animals, and (**D**) MMSE. The correlation values represent Spearman's Rho. Colored dots represent Clinical Dementia Rating (CDR) 0 (black) and CDR 0.5 (orange). MDT-OS, Mnemonic Discrimination Task for Objects and Scenes; RAVLT, Rey Auditory Verbal Learning Test; MMSE, Mini-Mental State Examination; PACC5, Preclinical Alzheimer's Cognitive Composite.

As part of the validation, we investigated the task's construct validity regarding conventional cognitive measures (see Table 4 and Figures 4–6). The relationships were statistically significant and positive (i.e., better mobile app-based memory performance was associated with better performance on the conventional cognitive tests). ORR-LDR and MDT-OS were weakly to moderately correlated with the PACC5 (*r* = 0.44, *p* < .001, and *r* = 0.32, *p* < .001). ORR-LDR showed weak to moderate relationships with PACC5 subtests RAVLT delayed recall (*r* = 0.40, *p* < .001) and Digit-Symbol-Coding (*r* = 0.28, *p* = 0.008). MDT-OS was weakly associated with the PACC5 subtest RAVLT delayed recall (*r* = 0.32, *p* < .001).

**Table 4:** Cognitive measures and correlation to mobile app-based measures.

Cognitive measures (PACC5)	Cognitive domain	Correlation ORR-LDR	Correlation MDT-OS
PACC5 composite	Multi-domain	*r* = 0.44, *p* < .001*	*r* = 0.32, *p* < .001*
MMSE	Multi-domain	*r* = 0.06, *p* = .609	*r* = 0.03, *p* = .724
RAVLT delayed recall	Memory	*r* = 0.40, *p* < .001*	*r* = 0.32, *p* < .001*
Digit-Symbol-Coding	Executive	*r* = 0.28, *p* = .008*	*r* = 0.14, *p* = .136
Word fluency animals	Executive	*r* = 0.19, *p* = .060	*r* = 0.07, *p* = .418
TMT A	Speed/attention	*r* = 0.21, *p* = .039*	*r* = 0.08, *p* = .377
TMT B	Executive	*r* = 0.10, *p* = .355	*r* = 0.21, *p* < .020*
Digit span	Speed/attention	*r* = 0.15, *p* = .165	*r* = 0.17, *p* = .077
Block Design	Visuospatial	*r* = 0.21, *p* = .060	*r* = 0.28, *p* = .003*
Word fluency FAS	Executive	*r* = 0.18, *p* = .078	*r* = 0.14, *p* = .129
Stroop	Executive	*r* = 0.39, *p* < .001*	*r* = 0.26, *p* = .005*
RAVLT learning	Memory	*r* = 0.27, *p* = .010*	*r* = 0.33, *p* < .001*
BVMT learning	Memory	*r* = 0.26, *p* = .060	*r* = 0.17, *p* = .175
BVMT delayed recall	Memory	*r* = 0.37, *p* = .007*	*r* = 0.19, *p* = .122

The correlation values represent Spearman's Rho.

ORR-LDR, Object-in-Room Recall Task; MDT-OS, Mnemonic Discrimination Task for Objects and Scenes; PACC5, Preclinical Alzheimer's Cognitive Composite; MMSE, Mini-Mental State Examination; RAVLT, Rey Auditory Verbal Learning Test; TMT, Trail Making Test; BVMT, Brief Visuospatial Memory Test—Delta version.

* *p*<.05.

Next, we explored relationships between mobile app-based memory tasks and all conventional cognitive measures separated by cognitive domains (episodic memory, speed and attention, executive function, visuospatial function). For episodic memory measures, weak to moderate correlations were observed with ORR-LDR (*r* = 0.27–0.40) and MDT-OS (*r* = 0.32–0.33). A weak correlation was seen with ORR-LDR (*r* = 0.21) for speed and attention, while MDT-OS showed no significant correlation. Weak to moderate correlations were observed between tests of executive function and ORR-LDR (*r* = 0.28–0.39) and MDT-OS (*r* = 0.21–0.26). For visuospatial functioning, a weak correlation was seen in relation to MDT-OS (*r* = 0.28).

As mentioned in the methods section, the time between follow-up in-clinic examination and the initial mobile app-based test sessions differed across participants (mean = 11.19 months, SD = 6.55 months, range = 0.34–21.49 months). To explore whether this affected the results, linear regressions were performed using the time elapsed and conventional cognitive tests as predictors and mobile app-based tasks as an outcome. The model showed no significant associations for time elapsed, indicating that the time elapsed between the examinations did not significantly affect the correlation between mobile app-based tasks and any of the conventional cognitive tests.

## Discussion

In this study, we utilized remote and unsupervised mobile app-based cognitive testing in a sample of older adults. We set out to explore important aspects to consider when employing unsupervised testing, including contextual factors, device-specific factors, and individual traits. In our sample, we found that these factors had very limited influence on task performance. Our participants rated the tasks as challenging but highly interesting at the same time. Importantly, distractions were not associated with actual test performance outcomes. We observed good psychometric properties, and test-retest reliability for one session was initially poor-to-moderate but increased significantly when using the average of two sessions. Lastly, the mobile app-based cognitive measures demonstrated construct validity concerning meaningful in-clinic cognitive measures—including the PACC5.

To investigate whether the outcomes of remote and unsupervised digital cognitive assessments are comparable to traditional cognitive measures, construct validity was determined. This is usually accomplished by comparing novel measures against conventional cognitive tests used in controlled studies (14, 19, 21, 48–50). Our results showed that, as expected, the correlation between mobile app-based memory tasks and individual conventional cognitive measures was strongest for episodic memory measures, indicating convergent validity. In addition, they were related to tests of timed executive function with weaker relationships to other measures (discriminant validity).

A cognitive measure often used in the early stages of AD is the PACC, and earlier studies have already compared unsupervised cognitive assessments against the PACC in similar samples. For example, a composite measure of the Boston Remote Assessment for Neurocognitive Health (BRANCH) (19) was strongly correlated with the PACC5 (*r* = 0.62) in cognitively normal participants. Another recent study (34) used a composite of similar but not identical tasks as used in this study and demonstrated a strong correlation to PACC (*r* = 0.51) in cognitively normal individuals without cognitive complaints. A study using the Ambulatory Research in Cognition app (15) in primarily cognitively normal participants (again using a composite of cognitive measures) reported a strong correlation with a global composite similar to the PACC (*r* = −0.53). Our study found that our mobile app-based tasks were significantly associated with the PACC5 (*r* = 0.3–0.5), with similar effect sizes reported in previous work (15, 19, 34). However, our study did not use a composite measure but individual memory scores. As expected, they were most strongly associated with conventional memory measures (*r* = 0.27–0.40), which align nicely with relationships of pure memory measures in the abovementioned studies (*r* = 0.30–0.47 and −0.22–0.32) (15, 19). Thus, our results support the notion that the mobile app-based memory measures assess relevant cognitive functions as confirmed by conventional cognitive outcomes but also showed associations with speed and executive function. It is important to note that construct validity may prove initial validation for newly developed cognitive tests. However, traditional cognitive tests only weakly correlate with AD biomarkers in preclinical stages. For example, the cross-sectional association between amyloid-β and conventional cognitive tests in biomarker-defined preclinical AD is generally weak (51–54). Thus, the possible usefulness for specific disease-related applications may not primarily depend on the correlation with conventional in-clinic tests (1). Furthermore, the correlation between conventional cognitive tests measuring similar cognitive domains does not consistently correlate strongly. For example, in our sample, the correlation between the memory measures RAVLT delayed recall and BVMT delayed recall correlated only moderately (*r* = 0.39, *p* < .001).

If a task is too difficult, it is less useful for individuals with more impaired cognitive function (floor effect)—the reverse is true when a task is too easy (ceiling effect). We did not find apparent floor or ceiling effects in our study population. However, for ORR-LDR, 10% (*n* = 9) of participants scored below chance which indicates that this specific delay period (up to 72 h) might have been too difficult for some of the participants of this old age sample. Future studies utilizing this type of unsupervised and remote long-term delayed recall should thus implement appropriate measures to strengthen study adherence or limit the delay time to shorter time periods.

A challenge in administering long-term delayed recall tests is that the test leader needs to contact the participant several times over a period. This has traditionally been solved by calling the participants the next day and asking them what they remember from a list of words for example. The use of mobile app-based tools has simplified this. In our study, we remind participants using a push notification on their phone, provided they have chosen to accept push notifications, and ask them to log in the next day. However, a significant proportion of participants performed the long-term delayed recall (ORR-LDR) after the recommended time of 24 h. While only 8% of test sessions had not been completed at all, many (35%) were simply completed too late. This could be because push notifications were not activated, they were not noticed, or because the participant was occupied elsewhere the following day. Although many participants completed the long-term delayed recall too late, our analysis showed that the proportion of data with increased delayed recall times outside the schedule was not higher in participants with CDR 0.5 as compared to those with CDR 0. This suggests that the schedule is as feasible for participants with CDR 0.5 as those with CDR 0, boding well for implementing this measure in clinical groups (for example patients with mild cognitive impairment).

One possibility to strengthen task completion within the given time is to improve the clarity of the task instruction. While the instructions were not detailing that the participants should carefully follow the recommended time, we recently optimized the instructions by adding clarifications regarding the importance of the timing of the delayed recall. This is particularly relevant in long-term delayed recall settings with delay times that extend to the next day. Before the participants initiate the task, they are now clearly informed that they should ensure availability on the next day to continue the task (“Tomorrow you will be asked about your memory of today's task. Before you start today's task, please make sure that you are available for the next task at about the same time tomorrow.”). It now needs to be evaluated whether this will improve adherence to the study design in future studies.

Given the importance of adherence in unsupervised studies, the participants' experience of usability and tolerability is especially important. The tasks were generally perceived as interesting in our study and lower task interest was not associated with actual task performance. We also collected information about distractions and concentration during the tasks, important confounders in an unsupervised test setting. Few participants reported being distracted, and most could focus well during tasks. This indicates that most participants can find an environment where they can perform the tasks undisturbed. While it still remains unclear what disturbances did appear during the test sessions, our analyses showed that experienced distractions and concentration did not affect task performance. One rationale for using mobile app-based tools is that cognitive data can be collected unobtrusively compared to a clinic visit, thus reducing participant burden. Most participants rated the tasks as rather challenging. Challenging tasks might be beneficial following the idea that sensitive cognitive tests likely need to be demanding for the participant to allow for the detection of subtle cognitive impairment in the preclinical phase of AD (55). However, how this will be associated with long-term participation over time (23) will need to be explored. For future practical implementation it holds promise that most of the participants in our study rated the tasks as interesting.

For future clinical implementation, sufficient test-retest reliability is critical for a test assessing cognitive performance over time. We initially reported poor–moderate test-retest reliability using a single test session for our sample. However, when using the mean across two sessions, test-retest reliability increased to moderate–good. Note that reliability was challenging for a single session of ORR-LDR. As reported above, ORR-LDR showed increased variability in the time between encoding and retrieval within and across participants which was in turn associated with task performance. Thus, variability in time between encoding and retrieval is likely an important limitation for test-retest reliability in this case and may increase in future studies with restricted delay periods. The generally lower retest reliability for one test session compared to the average of two test sessions is expected and consistent with earlier work on short unsupervised memory tests *via* mobile app-based approaches (14, 56). Test-retest reliability for a single session in our study was lower compared to conventional cognitive testing (57). However, the increased retest reliability for an average of two sessions is equal to or better than what has been reported for conventional cognitive testing as well as computerized testing (58). Given that one advantage of mobile app-based approaches is that cognitive tasks can be administered repeatedly, we conclude that cognitive data in such settings should be collected at several time points to obtain significantly higher test-retest reliability.

Our study had several important limitations. First, our sample was Swedish-speaking and very limited in terms of cultural and language diversity. Future studies should attempt to include cohorts with a more diverse background. Second, it is essential to point out that this study was an experimental study conducted in a convenience sample drawn from a larger population-based study. Thus, we cannot rule out potential biases in recruitment that might limit the generalizability of our results. For example, persons that are cognitively healthier might be more likely to consent to app-based studies. Comparisons between those recruited to the app-based study and those that so far have not been enrolled showed a significant effect where the group that was enrolled in the mobile app study was slightly better educated and showed slightly higher global cognitive scores. This effect was, however, minimal, and the groups did not differ regarding clinical rating or sex ratio. Thirdly, some participants did not strictly adhere to the study design (i.e., completing the long-term delayed recall test after the recommended time limit), resulting in missing data for a proportion of the participants. Fourth, the present limited recruitment is also a potential limitation (i.e., 172 of 692 have signed the informed consent and initiated the testing). However, we anticipate that this number will increase as the study continues. Lastly, the study is so far missing AD biomarkers, including longitudinal CSF- and neuroimaging-based biomarkers and clinical progression.

In conclusion, this study has explored the validity and retest reliability of cognitive assessments obtained in unsupervised environments which is critical to initiate cognitive testing outside research centers on a large scale. Continued studies in the H70 cohort will investigate the mobile app-based tasks in relation to AD biomarkers and clinical progression, address the potential issues of long-term adherence and report more comprehensive experiences from participants.

## Data Availability

Due to the nature of this research, participants of this study did not agree for their data to be shared publicly, so supporting data is not available. Requests for data will be considered by the H70 study data sharing committee on the basis of scientific priorities and overlapping interests. Requests to access these datasets should be directed to Ingmar Skoog (ingmar.skoog@gu.se).
